# An Antioxidant Dietary Supplement Improves Brain-Derived Neurotrophic Factor Levels in Serum of Aged Dogs: Preliminary Results

**DOI:** 10.1155/2015/412501

**Published:** 2015-06-23

**Authors:** Sara Sechi, Francesca Chiavolelli, Nicoletta Spissu, Alessandro Di Cerbo, Sergio Canello, Gianandrea Guidetti, Filippo Fiore, Raffaella Cocco

**Affiliations:** ^1^Department of Veterinary Medicine, Pathology and Veterinary Clinic Section, Via Vienna 2, 07100 Sassari, Italy; ^2^SANYpet S.p.a., Research and Development Department, Via Austria 3, Bagnoli di Sopra, 35023 Padua, Italy; ^3^School of Specialization in Clinical Biochemistry, “G. d'Annunzio” University, Via dei Vestini 31, 66100 Chieti, Italy

## Abstract

Biological aging is characterized by a progressive accumulation of oxidative damage and decreased endogenous antioxidant defense mechanisms. The production of oxidants by normal metabolism damages proteins, lipids, and nucleotides, which may contribute to cognitive impairment. In this study 36 dogs were randomly divided into four groups and fed croquettes of different compositions for 6 months. We monitored derivatives of reactive oxygen metabolites (dROMs) and biological antioxidant potential (BAP) levels in dogs' plasma samples as well as brain-derived neurotrophic factor (BDNF) serum levels at the beginning and at the end of the dietary regime. Our results showed that a dietary regime, enriched with antioxidants, induced a significant decrease of plasma levels of dROMs (*p* < 0.005) and a significant increase in BDNF serum levels (*p* < 0.005) after six months. Thus, we hypothesized a possible role of the diet in modulating pro- and antioxidant species as well as BDNF levels in plasma and serum, respectively. In conclusion the proposed diet enriched with antioxidants might be considered a valid alternative and a valuable strategy to counteract aging-related cognitive decline in elderly dogs.

## 1. Introduction

Biological aging is characterized by a progressive accumulation of oxidative damage and decreased endogenous antioxidant defense mechanisms [1]. The production of oxidants by normal metabolism damages proteins, lipids, and nucleotides which may contribute to neurodegeneration and, subsequently, cognitive impairment such as Alzheimer's (AD) and Parkinson's diseases (PD) [2, 3]. Although the body normally has sufficient protection producing endogenous antioxidants enzymes, such as catalase, glutathione and superoxide dismutase, an imbalance in the pro-oxidant/antioxidant species could increase the risk for lipid peroxidation DNA and protein damage. Oxidative damage also affects neuron function and may contribute to a cognition decline with age. The excess of reactive oxygen species in neuronal level can lead to irreversible damage of cytoskeleton and the microtubular network [4]. Microtubules, important dynamics polar formations of the cytoskeleton, are abundant in neurons, where they provide a scaffold also for their dendrites. Because of oxidative stress conditions the peroxynitrite may react with tyrosine to form 3-nitro-L-tyrosine (3NT). This final product may be selectively incorporated into the *α*-tubulin, resulting in an irreversible blocking of the characteristic dynamics of microtubules causing morphological changes, neuronal death and consequent onset of neurodegenerative diseases. Several experimental evidences have shown the importance of polyunsaturated fatty acids (PUFAs) in human and animal nutrition and development [5]. Further, an omega-3 and omega-6 deficiency likely correlates with the development of behavioral disorder [6].

Thus we hypothesized that a valid alternative and valuable strategy to counteract aging-related cognitive decline and neurodegeneration in aged dogs might be a dietary supplementation with antioxidants. Recent studies of animal models of neurodegenerative diseases suggest that dietary restriction can increase neurons endurance to age-related and disease-specific stresses [7]. Neurotrophic factors, including nerve growth factor (NGF) and brain-derived neurotrophic factor (BDNF), can protect neurons against death, as observed for* in vivo* and* in vitro* models of acute (stroke, trauma, and seizures) and chronic (Alzheimer's and Parkinson's diseases) neurodegenerative diseases [8].

Our interest in the neurological effects of age stems from a more general concern with the development of a canine model of human cognitive aging. Dogs show age-related pathology similar to that observed in elderly humans, like learning and development impairments [9]. Although the exact mechanisms have not been established so far, recent evidences indicate that a combination of an antioxidant-enriched diet with essential fatty acids, like omega-3 and omega-6, can be used to reduce age-dependent impairments and cognitive decline in aged dogs [10]. We focused on BDNF due to its role in supporting the survival and growth of many neuronal subtypes and in mediating the synaptic efficacy, neuronal connectivity and plasticity [11].

We hypothesized that BDNF serum levels might be modulated by an antioxidant-enriched diet.

In humans, BDNF mRNA and protein are decreased in the cortex and hippocampus in mild cognitive impairment (MCI) and AD [12] and are also decreased in cognitive decline [13]. In animals, decreased BDNF serum levels result in a LTP and memory deficit, thus increasing BDNF availability in the brain may be a viable strategy to counteract cognitive decline with aging.

The canine model provides us with an opportunity to test the relationship between cognitive impairment in aging, related to a decrease in BDNF serum levels, and the effects of non-pharmacological interventions.

The aim of this study was to evaluate the effects of a novel dietary supplement endowed with antioxidant properties as an adjuvant in the prevention of oxidative stress conditions and neurodegenerative disorders in aged dogs. The supplement consisted in a mixed formula of fished or chicken proteins, rice carbohydrates,* Grifola frondosa*,* Curcuma longa*,* Carica papaya*,* Punica granatum*,* Aloe vera*,* Polygonum cuspidatum*,* Solanum lycopersicum*,* Vitis vinifera*,* Rosmarinus officinalis* and an Omega 3/6 ratio of 1 : 0.8.

*Grifola Frondosa* is a culinary-medicinal mushroom that may play an important role in the prevention of many age-associated neurological dysfunctions, including Alzheimer's and Parkinson's diseases. The mushroom shows neuroprotective, antioxidant and anti-(neuro)inflammatory effects; in fact it reduces beta amyloid-induced neurotoxicity, neurite outgrowth stimulation, and nerve growth factor (NGF) synthesis [14]. It can be considered a useful therapeutic agent in the management and/or treatment of neurodegenerative diseases [15]. It was suggested that there could be an overall improvement in cognitive abilities of subjects when incorporated in their daily diet [16].

*Curcuma Longa* is a naturally occurring phytochemical compound endowed with powerful free radical scavenging activity [17]. Farinacci et al. observed that curcumin, polyphenol derived from* Curcuma longa*, reduced neutrophils adhesion and superoxide production* in vitro* [18]. Moreover, it resulted beneficially in improving spatial attention and motivation deficits associated with impaired cognition in aging and AD, in both humans and dogs [19]. Head et al. observed that 9 aged beagles provided with a medical food cocktail containing 95% of curcuminoids for 3 months had significantly lower error scores and were more accurate across all distances, suggesting an overall improvement in spatial attention [20]. da Rocha et al. demonstrated that* C. longa* had neuroprotective effects* in vitro* and* in vivo* by targeting biochemical pathways associated with neurodegenerative disorders that include cognitive impairments, energy/fatigue, mood and anxiety [21, 22].

*Carica papaya* is a natural compound with a high phenolic and flavonoid content which explains its free radical scavenging and antioxidant potential [22–24]. Mehdipour et al. demonstrated its antioxidant effect* in vitro* and reported a significant decrease in blood lipid peroxidation, while the blood total antioxidant power resulted significantly increased (*p* < 0.001) [24].

*Punica granatum* is a plant containing some species of flavonoids and anthocyanins, for example, delphinidin, cyaniding and pelargonidin, which have been shown to have an antioxidant activity* in vitro* [25] and* in vivo* [26]. The antioxidant action of* P. granatum* is related to its free radical scavenging activity against superoxide ions, mainly due to the presence of anthocyanins and to the ability to form metal chelates [25]. Rehydration effects have been also reported [27].

*Aloe vera* and its extracts have medicinal properties attributed to the active components such as anthrone, chromone, aloe verasin and hydroxylation [28, 29]. Sahu et al. demonstrated that* Aloe vera* had different degrees of antioxidant activity [30]. The antioxidant properties of this plant may depend on the radical scavenging activity. Moreover, life-long dietary supplementation of* A. vera* was shown to suppress many age-related consequences* in vivo*. Due to the presence of phenolic acids, polyphenols, sterols, fatty acids and indoles,* A. vera* may result to be effective in relieving symptoms associated with or preventing neurodegeneration [31].

*Polygonum cuspidatum* is an important natural source of resveratrol [32]. Due to its numerous pharmacological activities it has been used as an antioxidant [33]. Antioxidant activities of* P. cuspidatum* have been reported both* in vivo* and* in vitro* study [34]. Moreover, three of its dimers, parthenocissin A, quadrangularing A and pallidol, have shown strong free radical quenching and selective singlet oxygen scavenging activity [35]. Trans-resveratrol has been used as antidepressant in chronically stressed rats, probably by acting on the monoaminergic systems, such as the serotonergic and noradrenergic [33]. In fact, chronic treatment with trans-resveratrol was found to inhibit monoamine oxidase-A (MAO-A) activity in all the four brain regions, particularly in the frontal cortex and hippocampus [36].

*Solanum lycopersicum* is rich of vitamins C and E, lycopene, beta-carotene, lutein and flavonoids such as quercetin, phenols, ascorbic acid (AsA) and dehydroascorbic acid (DHA). It is considered an important plant able to prevent chronic diseases and improve energy balance and antioxidant activity [37]. Both direct and indirect antioxidant activity, as indicated by reduced malondialdehyde (MDA) and nitric oxide (NO) production and increased glutathione peroxidase (GPx) and superoxide dismutase (SOD) activity, support the conclusion that tomatoes containing anthocyanins can potentially provide better protection against oxidative stress related chronic diseases [38].

*Vitis vinifera* can be considered as a potential source of natural antioxidants, due to the presence of carotenoids such as lutein, beta-carotene and polyphenols [39]. The antioxidant activity of grape extracts is due to their reducing power [40]. The grape seed flavanol/procyanidin compounds may act as similar as reductones by donating electrons and reaching with free radicals to convert them to more stable products and terminating the free radical chain reaction [40]. Antioxidant activities of* V. vinifera* were also assessed by their capacity to prevent Fe^2+^-induced lipid peroxidation in microsomes and their action on Cu^2+^-induced lipid peroxidation in low-density lipoproteins [41]. Astringin, a stilbenoid present in* V. vinifera*, is endowed with an important antioxidant effect and a higher radical scavenger activity [41].

*Rosmarinus officinalis* is known to exert an antiproliferative, antioxidant and antibacterial activity [42]. The crude extract also has shown antioxidant and anti-inflammatory activities inhibiting NO production and reducing proinflammatory cytokines (IL-1*β*) and enzymes (COX-2) mRNA expression in LPS-activated cells thus highlighting its chemopreventive potential [43]. Afonso et al. showed that phenolic compounds from* R. officinalis* protected against hypercholesterolemia-induced oxidative stress, increasing the activities of antioxidant enzymes [44]. Several literature reports have demonstrated that* R. officinalis* exerted multiple benefits for neuronal system and alleviated mood disorders [45]. In particular its active compounds, luteolin, carnosic acid and rosmarinic acid, exhibited neurotrophic effects by improving cholinergic functions [46] and showed neuroprotective properties by inhibiting amyloid precursor protein synthesis and higher brain-derived neurotrophic factor production in hypothalamus cells [45]. Antidepressant-like effect of* R. officinalis* may be mediated by an interaction with the dopaminergic system, through the activation of dopamine D1 and D2 receptors [47].

Omega 3/6 fatty acids: a good balance of the Omega 3/6 fatty acids ratio in the food is a basic requirement to improve the inflammatory and neurological background [48]. More in detail, n-3 polyunsaturated fatty acids, usually found in fish oil, such as eicosapentaenoic acid (EPA) and docosahexaenoic acid (DHA), are known to both decrease the production of proinflammatory mediators and inhibit natural killer cell activity [49]. Moreover preclinical studies suggested that low plasma omega-6 and omega-3 fatty acids levels were associated with accelerated decline of peripheral nerve function with aging [50]. Intake of PUFAs, mainly omega-3 and omega-6, was shown to increase BDNF production in brain [50, 51]. Docking studies on PUFAs and their metabolites with BDNF revealed that PUFAs metabolites, mainly LXA_4, NPD1 and HDHA, had more binding affinity towards BDNF [51]. These metabolites of PUFAs are also responsible for modulation of BDNF activity [51].

## 2. Methods

### 2.1. Subjects

Thirty-six dogs of different breeds were randomly and equally divided into four groups based on age and diet (Table 1). First group, made up of 8 dogs (three males and five females, age 9 ± 0.08, mean ± standard error of the mean), was fed a control diet with organic chicken. Second group, made up of 8 dogs (three males and five females, age 7 ± 0.25, mean ± standard error of the mean), was fed a chicken-based food enriched with natural antioxidants. The third group, made up of 8 dogs (four males and four females, age 9 ± 0.63, mean ± standard error of the mean), was fed a fish-based meal and the fourth one was made up of 8 dogs (three males and five females, age 9 ± 0.94, mean ± standard error of the mean) and was fed a fished-based meal enriched with natural antioxidants.

d-ROMs and BAP tests (Free Radical Analytical System FRAS 4, H&D s.r.l., Langhirano PR, Italy) were performed before (T0) and at the end (T1) of the treatment in all animals in order to determine the oxidative stress status. The four diets were administered for a six-month period.

### 2.2. Sample Collection and Analysis

Blood samples were collected from each dog before the new dietary regime (T0) and at the end of the treatment (T1) after six months from cephalic vein and stored in two tubes, one with heparin and the other without anticoagulant. Heparinized plasma samples and serum samples were obtained by blood centrifugation at 4000 g× 1,5 min at 37°C.

The derivatives of reactive oxygen metabolites (dROMs) and the biological antioxidant potential (BAP), as indicators of oxidative stress, were measured by portable spectrophotometer (Free Radical Analytical System FRAS 4, H&D s.r.l., Langhirano PR, Italy) on plasma samples. In the dROMs test, reactive oxygen metabolites (primarily hydroperoxides) of the sample, in presence of iron released from plasma proteins by an acidic buffer, generate alkoxyl and peroxyl radicals, according to the Fenton reaction. Such radicals then oxidize an alkyl-substituted aromatic amine (N,N-dietylparaphenylendiamine), thus producing a pink-colored derivative which is photometrically quantified at 505 nm [52]. The dROMs concentration is directly proportional to the colour intensity and is expressed as U.CARR (Carratelli Units). One U.CARR corresponds to 0.8 mg/L hydrogen peroxide. The reference values of dROMS are summarized below:

- reference value 50–90 U.CARR;
- threshold borderline: 92–95 U.CARR;
- condition of mild oxidative stress: 100–120 U.CARR;
- condition of oxidative stress: 140–200 U.CARR;
- condition of strong oxidative stress: 220–300 U.CARR;
- strong oxidative stress: over 300 U.CARR.

In the BAP test plasma samples were added with a colored solution, obtained by mixing a ferric chloride solution with a thiocyanate derivative solution, which causes a discoloration, whose intensity is measured photometrically at 505 nm and it is proportional to the ability of the plasma to reduce ferric ions. The results are expressed as *μ*Mol/L of reduced ferric ions. Both tests were validated for canine species [53]. Range values of BAP are listed below:

- reference value 2000–4000 *μ*Mol/L;
- optimal values: >2200 *μ*Mol/L;
- threshold borderline: 2200–2000 *μ*Mol/L;
- discrete deficiency state: 2000–1800 *μ*Mol/L;
- deficiency state: 1800–1600 *μ*Mol/L;
- strong deficiency state: 1600–1400 *μ*Mol/L;
- very strong deficiency state: <1400 *μ*Mol/L.

### 2.3. BDNF Analysis

BDNF analysis was performed with BG BDNF ELISA kit (Blue Gene Biotech CO., LTD, Shanghai, China) designed for the quantitative determination of canine BDNF. The ELISA test reaction was performed using Crocodile mini Workstation (Totertek Berthold, Pforzheim, Germany). The kit utilizes a monoclonal anti-BDNF antibody and BDNF-HRP conjugate. The assay sample and buffer are incubated together with BDNF-HRP conjugate in precoated plate for one hour. After the incubation period, the wells are washed five times. The wells are then incubated with a substrate for HRP enzyme. The product of the enzyme-substrate reaction forms a blue colored complex. Finally, a stop solution is added to stop the reaction, which will then turn the solution yellow. The intensity of color is measured spectrophotometrically at 450 nm with a microplate reader. The intensity of the color is inversely proportional to the BDNF concentration since BDNF from samples and BDNF-HRP conjugate compete for the anti-BDNF antibody binding site. Since the number of sites is limited, as more sites are occupied by BDNF from the sample, fewer sites are led to bind BDNF-HRP conjugate. A standard curve is then plotted relating the intensity of the color (O.D) to the concentration of standards. The BDNF concentration of each sample is interpolated from this standard curve.

### 2.4. Statistical Analysis

All data are presented as the mean ± SEM. An unpaired 2-sample Student's *t*-test was used to compare the differences in plasma dROMs, BAP, and BDNF levels between the four groups. All statistical analyses were performed with GraphPad Prism 6 (GraphPad Software Inc., San Diego, CA, USA). *p* < 0.05 was considered significant.

## 3. Results

### 3.1. Oxidative Stress Status Evaluation

We firstly analyzed dROMs and BAP levels as a measure of the oxidative stress status of dogs. Analysis was made on dogs plasma in all of the four groups before starting the new dietary regime (T0) and at the end, after six months (T1).

**Figure 1:**
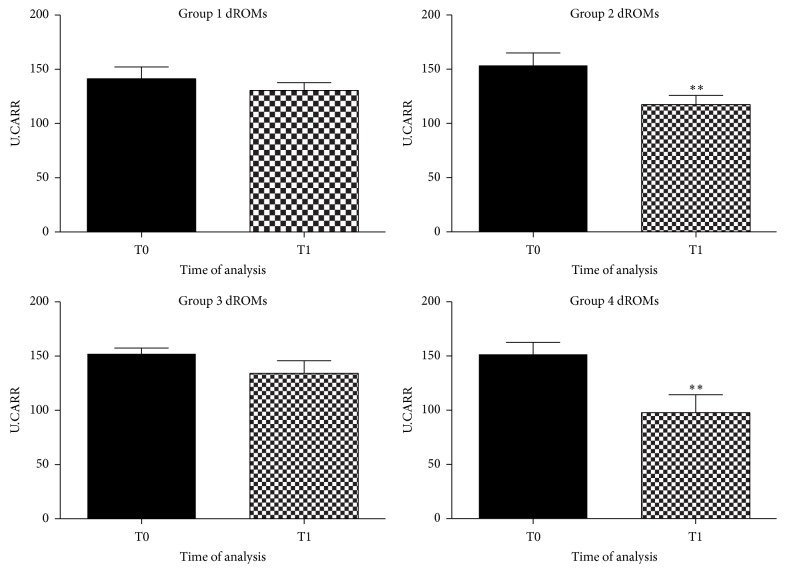
Graphical representation of dROMs in plasma of aged dogs before and after the 6 months of the dietary regime. A significant decrease of dROMs levels was observed in dogs fed with antioxidant supplementation, in the second group (^*∗∗*^ *p* < 0.005) and in the fourth group (^*∗∗*^ *p* < 0.005), respectively.

As shown in Figure 1, a significant decrease in plasma levels of dROMs, after six months of feeding regime, in groups 2 and 4 (food supplemented with antioxidant) was observed (*p* < 0.005). dROMs levels remained unaltered in the first (control group) and third groups (fish-based meal, without antioxidant addition).

These data indicate that a diet enriched with natural antioxidant might be able to promote a decrease of reactive species in plasma of aged dogs.

**Figure 2:**
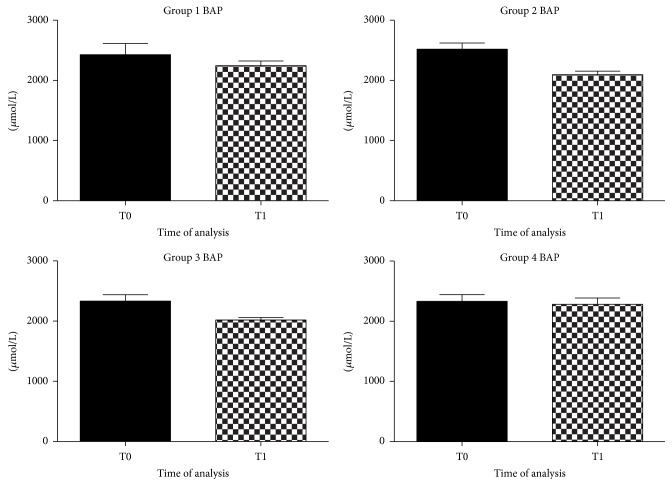
Graphical representation of BAP in plasma of aged dogs before and after 6 months of the dietary regime. BAP levels did not show significant modifications in all groups of dogs after the diet.

Antioxidant influence, evaluated with BAP analysis, remained unchanged in all groups of dogs after the diet (Figure 2).

Natural antioxidants seemed to modulate the balance between pro- and antioxidant species through the decrease of dROMs without increasing natural antioxidant defense.

### 3.2. BDNF Evaluation

Literature reports have shown a decrease in BDNF serum levels to negatively correlate with cognitive decline and deficits in LTP and memory in dogs [13]. BDNF is a neurotrophic factor that can protect neurons against death supporting the survival and growth of many neuronal subtypes [8]. An increase in BDNF serum levels is one of the factors underlying improvements in learning and memory [8, 54]. Increasing BDNF availability in the brain, by diet, may be a viable strategy to counteract cognitive decline with aging [7, 55, 56].

**Figure 3:**
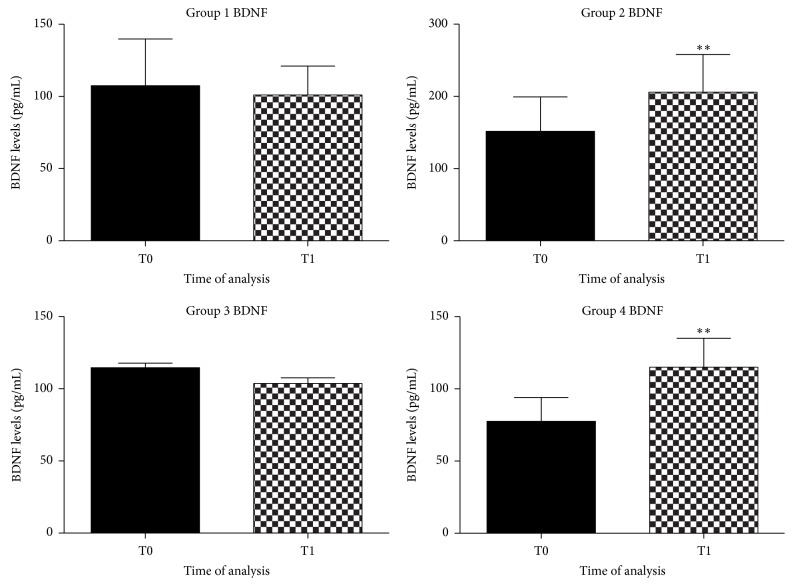
Graphical representation of BDNF in plasma of aged dogs before and after the 6 months of the dietary regime. A significant increase of BDNF levels was observed in dogs fed with antioxidant supplementation, in the second group (^*∗∗*^ *p* < 0.005) and in the fourth group (^*∗∗*^ *p* < 0.005), respectively.

We reported a significant BDNF serum levels increase in groups of dogs that received a diet enriched with antioxidant, *p* < 0.005 (Figure 3), while in the other groups BDNF serum levels remain unchanged.

## 4. Discussion

The purpose of this study was to evaluate the possible potential ability of a long-term dietary antioxidant supplementation in controlling the oxidative stress and the general health status of aged dogs. Moreover we demonstrated a possible modulation of BDNF serum levels by a diet with antioxidant supplementation.

Environmental stress and aging may induce psychological stress that possibly influences also the nutrition of pets. An unbalanced diet deficient in essential nutrients may represent a risk factor of degenerative diseases. Among the several mechanisms by which nutrients influence the health status, the balance of oxidative stress has a relevant role. Constantly, in the animal species, metabolic oxidative reactions take place. The goal of these reactions is to balance free radicals production with antioxidants molecules.

The inhibitory activity of antioxidant molecules was observed in* in vitro* studies using plants derivative compounds such as flavonoids, anthocyanins and other poliphenols evaluating their effects on the converting activities of *α*-amylase, *α*-glucosidase and angiotensin-converting enzyme (ACE). They all have shown an inhibitory activity on *α*-amylase, *α*-glucosidase and ACE [57].

Furthermore, a significant reduction in dROMs values in the experimental diet enriched with natural antioxidants was observed. In apparently healthy dogs, serum levels of the dROMs ranged between 50 and 90 U.CARR. These values were in agreement with those reported by Pasquini et al. [53]. The antioxidant supplementation significantly decreased dROMs levels from 155 U.CARR (T0) to 120 U.CARR (T1) in the second group and from 150 U.CARR (T0) to 95 U.CARR (T1) in the forth group. Differently, dogs fed the control diet, deficient in antioxidant nutrients, did not modulate the oxidative stress status. The antioxidant status revealed by BAP test was not affected by nutrition and values were at optimal levels throughout the observational period. Probably antioxidant supplements only affected dROMs species but not the endogenous antioxidant components of dogs, which remained in the initial optimal condition. This might be related to the really efficacy of the experimental diet in modulating the oxidative stress.

The antioxidant formulation employed in this experiment was based on* Grifola frondosa, Curcuma longa, Carica papaya, Punica granatum, Aloe vera, Polygonum cuspidatum, Solanum lycopersicum, Vitis vinifera *and* Rosmarinus officinalis* extracts.

All these compounds contain anthocyanins and polyphenols with antioxidant effects [7].

With this study we showed a decrease in dROMs species following the administration of the analyzed antioxidant formulation.

Our study reporting the scavenger activity of an antioxidant supplementation in dogs diet is in agreement with other studies about antioxidant effects of singular active principles included into an antioxidant supplementation [58]. The anthocyanins and polyphenols have antioxidant effects in some pathological conditions, such as metabolic disorders, aging-related diseases, cardiovascular diseases cancer, and inflammatory-related disturbs, as well as carotenoids and flavonoids [39, 59]. These compounds are inhibitors of lipid peroxidation probably by interfering with the glutathione activity [60].

Resveratrol (3,5,4′-trihydroxystilbene) is a polyphenol naturally present in grapes, berries, peanuts, and other vegetables [61], with therapeutic and neuroprotective functions [7]. Moreover trans-resveratrol, which is highly presented in* Polygonum cuspidatum*, could modulate BDNF levels through the monoaminergic system activation [36]. In our study we observed that a diet enriched with natural antioxidants was able to increase BDNF serum levels from 100 ± 0.5 pg/mL to 180 ± 0.8 pg/mL, while in the other groups remained unchanged. As to* Grifola frondosa*, an improvement in cognitive abilities in aged dogs was observed when adding* Curcuma longa* and* Aloe vera* to a daily diet through a stimulation of the BDNF synthesis [16]. According to what was observed by Sasaki et al., luteolin, carnosic acid, and rosmarinic acid, from* Rosmarinus officinalis*, exerted a neuroprotective activity probably modulating the neurotrophic metabolic pathway in the neuronal system [46]. Finally, Kumar et al. observed that PUFAs, like omega-3 and omega-6, along with their metabolites, had more binding affinity towards BDNF [51]. Further, the serum level increase of both fatty acids might be modulated by these metabolites which in turn could regulate the BDNF production in the brain. Neurotrophic factors, such as BDNF, can protect neurons against death and be a preventive approach in neurodegenerative conditions [7, 8].

By means of this new canine model of aging, we showed that providing antioxidants within a specific dietary supplement it was possible to restore the balance between pro- and antioxidants species, possibly modulating also BDNF serum levels. Future studies in both aged humans and dogs will be more effective if antioxidants combinations will be evaluated along with additional lifestyle improvements such as cognitive training and physical exercise.

## Figures and Tables

**Table 1:** Dogs' diet groups and their features.

Group	Pet food	Sex	Mean age ± SEM
1	Organic chicken	2M, 6F	9 ± 0.08
2	Chicken + antioxidants	5M, 3F	7 ± 0.25
3	Fish	6M, 2F	9 ± 0.63
4	Fish + antioxidants	3M, 5F	9 ± 0.94

Antioxidants added in groups 2 and 4 are *Grifola frondosa*, *Curcuma longa*, *Carica papaya*, *Punica granatum*, *Aloe vera*, *Polygonum cuspidatum*, *Solanum lycopersicum*, *Vitis vinifera*, and *Rosmarinus officinalis* an Omega 3/6 ratio of 1 : 0.8.
